# Severe postpartum hemorrhage and the risk of adverse maternal outcome: A comparative analysis of two population-based studies in France and the Netherlands

**DOI:** 10.1016/j.pmedr.2024.102665

**Published:** 2024-02-23

**Authors:** P.L.M. de Vries, C. Deneux-Tharaux, C. Caram-Deelder, F. Goffinet, D.D.C.A. Henriquez, A. Seco, J.G. van der Bom, T. van den Akker

**Affiliations:** a Department of Obstetrics, Leiden University Medical Centre, Leiden, The Netherlands; b Port-Royal Maternity Unit, Cochin Hospital, Assistance Publique-Hôpitaux de Paris, Paris, France; c Université Paris Cité, Inserm, Obstetrical, Perinatal and Paediatric Epidemiology Research Team (Epopé), CRESS UMR 1153, Paris, France; d Department of Clinical Epidemiology, Leiden University Medical Center, Leiden, The Netherlands; e Clinical Research Unit Necker Cochin, APHP, Paris, France; f Athena Institute, VU University, Amsterdam, The Netherlands

**Keywords:** Cross-country studies, Obstetric hemorrhage, Management, Clinical care, Severe maternal outcome, Maternal mortality, Maternal morbidity

## Abstract

**Objectives:**

Among women with severe PPH (sPPH) in France and the Netherlands, we compared incidence of adverse maternal outcome (major obstetric hemorrhage (≥2.5L blood loss) and/or hysterectomy and/or mortality) by mode of delivery. Second, we compared use and timing of resuscitation and transfusion management, second-line uterotonics and uterine-sparing interventions (intra-uterine tamponade, compression sutures, vascular ligation, arterial embolization) by mode of delivery.

**Methods:**

Secondary analysis of two population-based studies of women with sPPH in France and the Netherlands. Women were selected by a harmonized definition for sPPH: (total blood loss ≥ 1500 ml) AND (blood transfusion of ≥ 4 units packed red blood cells and/or multicomponent blood transfusion).

**Findings:**

Incidence of adverse maternal outcome after vaginal birth was 793/1002, 9.1 % in the Netherlands versus 88/214, 41.1 % in France and 259/342, 76.2% versus 160/270, 59.3% after cesarean. Hemostatic agents such as fibrinogen were administered less frequently (p < 0.001) in the Netherlands (vaginal birth: 83/1002, 8.3% versus 105/2014, 49.5% in France; cesarean: 47/342, 13.7% and 152/270, 55.6%). Second-line uterotonics were started significantly later after PPH-onset in the Netherlands than France (vaginal birth: 46 versus 25 min; cesarean: 45 versus 18 min). Uterine-sparing interventions were less frequently (p < 0.001) applied in the Netherlands after vaginal birth (394/1002,39.3 %, 134/214, 62.6%) and cesarean (133/342, 38.9 % and 155/270, 57.4%)**,** all initiated later after onset of refractory PPH in the Netherlands.

**Interpretation:**

Incidence of adverse maternal outcome was higher among women with sPPH in the Netherlands than France regardless mode of birth. Possible explanatory mechanisms are earlier and more frequent use of second-line uterotonics and uterine-sparing interventions in France compared to the Netherlands.

## 1 Introduction

Postpartum hemorrhage (PPH) is one of the leading causes of severe maternal outcomes globally and has recently been on the rise in several high-income countries (Corbetta-Rastelli et al., 2023, Reale et al., 2010, Givens et al., 2022). Between countries of this type, marked variations in maternal outcomes after PPH have been reported (Kallianidis et al., 2020, Diguisto and Saucedo, 2022). These are not likely to be explained by individual patient characteristics, differences in mode of delivery or by different strategies of PPH-prevention and initial PPH-management between high-income countries (Kallianidis et al., 2021, McCall et al., 2021). This raises the question whether differences in clinical management of refractory PPH may be involved. Such differences might be compounded by a lack of robust evidence for the management of severe PPH and differences in clinical guidance between countries (de Vries et al., 2023).

Peripartum hysterectomy is a last-resort management option in case of severe PPH. Earlier studies reported a PPH-related maternal mortality ratio and peripartum hysterectomy rate nearly 2-fold as high in France as compared to the Netherlands (Kallianidis et al., 2020, Diguisto and Saucedo, 2022, van den Akker et al., Ramler et al., 2022, Saucedo and Deneux-Tharaux, 2021). Yet, more than half of PPH-related maternal deaths in the Netherlands happened in women with the uterus still in place, suggesting important delay in PPH-management (Ramler et al., 2022). The importance of timely escalation to more invasive management options for PPH has been stressed by several authors (Henriquez et al., 2018, Della Torre et al., 2011, Lepine et al., 2020). However, the optimal timing of interventions between the onset of PPH in relation to the total amount of blood loss or severe maternal outcome has not been well defined (Howard and Grobman, 2015).

Intercountry comparisons of pregnancy outcomes have shown to be useful in revealing suboptimal care by scrutinizing the specificities of different national contexts (de Vries et al., 2023). As clinical practice and maternal outcome of postpartum hemorrhage varies substantially between France and the Netherlands, we hypothesized that a comparison of the severe PPH-management strategies between these two countries could contribute to a better understanding of optimal PPH-management and the reported differences in terms of severe maternal outcome.

The primary outcome of this study was to compare the incidence of adverse maternal outcome, defined as a composite of major obstetric hemorrhage (≥2.5L of blood loss and/or hysterectomy and/or mortality) among women suffering equally severe PPH in France and the Netherlands by mode of delivery. Second, we aimed to compare use and timing of resuscitation and transfusion management, second-line uterotonics and uterine-sparing interventions between both countries by mode of delivery.

## 2 Materials and methods

### 2.1 Design

Secondary analysis of two population-based studies.

### 2.2 Source and study population

For the Netherlands, data were selected from the TeMpOH-1 study, a nationwide retrospective cohort study of women with severe PPH recruited in 61 hospitals in the Netherlands from January 2011 to January 2013. For France, data were extracted from the EpiMOMs study, a population‐based prospective study designed specifically to study severe maternal morbidity in six French regions between May 2012 and November 2013 that collected data from 119 public and private maternity units.

A harmonized definition of severe PPH was applied to select from both studies the broadest common study-population (figure S1). We defined severe PPH as: (total blood loss ≥ 1500 ml) and (blood transfusion of ≥ 4 units of packed red blood cells AND/OR multicomponent blood transfusion). A multicomponent blood transfusion was defined as blood transfusion consisting of a combination of red blood cells and fresh frozen plasma and/or platelet concentrates.

From this study population, we selected women with ‘refractory PPH’, which pertained to having severe PPH according to our harmonized definition, and which was refractory to first-line management (uterine massage, exploration of the uterine cavity, assessment of the genital tract and administration of oxytocin).

### 2.3 Data collection

Women in the TeMpOH‐1 study were considered eligible for inclusion in the cohort by cross‐referencing data from hospitals’ blood transfusion services with local birth registers in participating hospitals. Women in the EpiMOMs study were identified prospectively by caregivers in participating hospitals and validated by a review of birth logbooks and registers, hospital discharge databases, and laboratory records. For both studies, details pertaining to data collection have been described elsewhere (Siddiqui et al., 2019, Henriquez et al., 2019).

PPH in France and the Netherlands was managed according to the national guidelines applicable at the time in both countries (NVOG, 2018, Goffinet et al., 2005). An overview of both guidelines is given in Table S1.

Blood loss measurement in the TeMpOH‐1 study was obtained by weighing gauzes, cloths and surgical swabs and by suction canisters or collector bags in EpiMOMs. Data dictionaries were provided by each country. If a certain variable was not available, we sought to create a new variable as long as it was comparable in both datasets. If the equivalent of a variable could not be identified in one of the two databases, it was excluded or presented with a dash. Variables not matching after mapping due to different coding, were subjected to harmonized coding. Availability and comparability of each respective dataset are presented in Table S2.

From both databases, we abstracted variables regarding patient characteristics, etiology of bleeding, initial PPH-management, resuscitation- and transfusion management, second-line uterotonics, obstetric management and maternal outcome. Adverse maternal outcome was defined as a composite of bleeding ≥ 2.5L, hysterectomy or mortality. For the subgroup of women with refractory PPH, we assessed the association between time of onset of the first uterine-sparing intervention (one of the following: intra-uterine balloon tamponade, embolization, compression sutures, vascular ligation) and total volume of blood loss. If multiple uterine-sparing interventions were performed, time of onset of the first uterine-sparing intervention in the sequence was considered as timing of intervention. Patient characteristics were assessed for overall birth and per mode of delivery. All other variables were stratified by mode of delivery.

### 2.4 Statistical analyses

Incidence of severe PPH was calculated per 1,000 births and presented with a 95 % confidence interval. Data were checked for normal distribution by histograms. Categorical data were presented by frequency and percentage, and continuous variables by median and interquartile range (25th to 75th percentile) and by mean and standard deviation. Statistical testing by Chi-square tests, T-tests, and kruskall Wallis test to test our null-hypothesis that there is no difference between the France and the Netherlands with regard to the assessed variables. Missing data can be consulted in the supporting information (Table S3). The total volume of blood loss as a function of the timing of the first invasive intervention was displayed in scatterplots. All analyses were conducted using STATA v15.

Ethical approval: The TeMpOH‐1 study was approved by the ethics committee of the Leiden University Medical Center on 31 January 2013 (P12.273) and by the institutional review board of each participating hospital. The study was registered in the Netherlands Trial Register (NTR4079). Need to obtain informed consent was waived by the ethics committee. The EpiMOMs study was approved by the appropriate institutional review board, the Commission Nationale de l’Informatique et des Libertés (CNIL, number 912210). Need to obtain informed consent was waived, according to the French legislation at that time. Women included in the study were informed and did not indicate their opposition to participate.

## 3 Results

### 3.1 Patient and hemorrhage characteristics

In Table 1 we describe the patient characteristics of women sustaining severe PPH in France and the Netherlands. As compared to the Netherlands, more women with severe PPH who gave birth vaginally had an assisted birth in France (212/1002, 21.2 % versus 62/214, 28.9 %) or cesarean (342/1344, 25.5 % versus 270/484, 55.8 %). In the Netherlands, more women had a history of PPH regardless mode of birth (vaginal birth:144/1002, 25.1 % versus 15/214, 12.9 %, p < 0.001 and for cesarean: 41/342, 17.2 % versus 12/270, 7.8 %, p < 0.001). In addition, more women in the Netherlands suffered from hypertensive disorders than in France after vaginal birth (96/1002, 9.6 % versus 9/214, 4.2 %, p = 0.01 but not after cesarean birth (39/342, 11.4 % versus 27/270, 10.1 % respectively, p = 0.58) (Table 1). Main causes of severe PPH were similar among both countries (Table S4).

**Table 1:** Patient and birth characteristics among women with severe PPH for overall births and stratified by mode of birth in France and the Netherlands (2011–2013).

		France	The Netherlands				
Population based denominator		182,309	207,101				
Number of women with sPPH^1^		484	1344				
Incidence sPPH per 1000 births		2.7(2.4–2.9)	5.0 (4.7–5.2)				

		Overall births			Vaginal births					Cesarean births				
	France		The Netherlands		France		The Netherlands			France		The Netherlands		
					N = 214		N = 1002		P-value	N = 270		N = 342		P-value
		N = 484	N = 1344		n	(%)	n	(%)		n	(%)	n	(%)	
Patient characteristics														
Age (Mean (std))^2^					30.7	(5.1)	31.2	(4.9)	0.15	32.9	(5.5)	33	(5.1)	0,7
Parity									0.07					0,06
*nulliparity*	210	(43.4)	532	(39.6)	97	(45.5)	428	(42.7)		112	(42.4)	104	(30,4)	
*multiparous, no previous cesarean*	177	(36.6)	621	(42.2)	95	(44.6)	493	(49.2)		82	(53.2)	128	(53.4)	
*multiparous with previous cesarean*	93	(19.2)	191	(14.2)	21	(9.9)	81	(8.11)		72	(46.8)	110	(46.2)	
History of PPH	27	(10.0)	185	(22.7)	15	(12.9)	144	(25.1)	< 0,001	12	(7.8)	41	(17.2)	< 0,001
Multiple pregnancy	58	(12.0)	84	(6.3)	14	(6.5)	46	(4.6)	0.26	44	(16.3)	38	(11.1)	0,06
Hypertensive disorder	36	(7.4)	135	(10.0)	9	(4.2)	96	(9.6)	0,01	27	(10.0)	39	(11.4)	0,58
Macrosomia	75	(15.5)	280	(20.8)	35	(16.4)	213	(21.3)	0,11	38	(14.1)	67	(19.6)	0,07
Characteristics of birth														
Weeks gestational age (IQR^3^)	39	(37–41)	39	(38–71)	39	(38–41)	39		0,43	37	(36–68)	37	(35–39)	0,36
Induction of labor	119	(32.3)	474	(39.6)	68	(31.8)	398	(39.7)	0,06	51	(18.9)	76	(38.9)	0,33
Mode of birth *Spontaneous vaginal birth* *Assisted birth* *Elective cesarean* *Emergency cesarean*	152 62 118 152	(31.4) (12.8) (24.4) (31.4)	790 212 147 195	(58.8) (15.8) (11.0) (14.5)	152 62 - -	(71.0) (28.9)	790 212 - -	(78.8) (21.2)	< 0.001	- - 118 152	(43.7) (56.2)	- - 147 195	(43.0) (57.0)	0.15
Prophylactic uterotonics^4^	432	(89.3)	1204	(89.6)	198	(92.5)	908	(90.6)	0.38	234	86.6	296	86.6	0.97

^1^ = severe postpartum hemorrhage ^2^ = standard deviation ^3^ = inter quartile range ^4^ = Oxyotocin 5 or 10 IU intramuscular or slow intravenous in both countries

As published in the TeMpOH-1 study, the incidence of severe PPH according to our harmonized definition in the Netherlands was 5.0 per 1000 livebirths (1344/270,101). This is in comparison to the EpiMOMs study in France, which found an incidence of severe PPH of 2.7 per 1,000 livebirths (488/182,309) (Figure S1, Table 1).

### 3.2 Adverse maternal outcome

Adverse maternal outcome was significantly more prevalent among women with severe PPH in the Netherlands as compared to women with severe PPH in France regardless mode of birth (vaginal birth: 793/1002, 79.1 % and 88/214, 41.1 %, p < 0.001) and cesarean: (259/342, 76.2 % versus 160/270, 59.3 % respectively, p < 0.001). Major obstetric hemorrhage was more prevalent among women with severe PPH in the Netherlands as compared to women in France both in case of vaginal birth (792/1002,79 % versus 82/214,12.2 %, p < 0.001) and cesarean (257/342, 75.6 % versus 142/270, 52.6 %, p < 0.001). Peripartum hysterectomy was significantly less frequently performed in the Netherlands than France after vaginal birth (27/1002,2.7 % versus 26/214,12.2 %, p < 0.001) and cesarean (46/342, 13.5 % versus 55/270, 20.4 %, p = 0.02). We do not report any significant differences in terms of maternal mortality (Table 2). The profile of the women who died from severe PPH in both countries is presented in table S5.

**Table 2:** Univariate analysis. Comparing incidence of adverse maternal outcome in women with severe PPH stratified by mode of birth between France and the Netherlands (2011–2013).

Vaginal birth Cesarean birth										
	**France**		**The Netherlands**			**France**		**The Netherlands**		
	N = 214 n	%	N = 1002 n	%	P-value	N = 270 n	%	N = 342 n	%	P-value
Total volume blood loss (L) ^1+2^	2	(1.6–2.5)	3	(2.5–4.0)	<0.001	2	(1.7–3.0)	3	(2.5–4.0)	<0.001
Blood loss ≥ 2,5 L	82	(38.3)	792	(79.0)	<0.001	142	(52.6)	257	(75.6)	<0.001
Hysterectomy	26	(12.2)	27	(2.7)	<0.001	55	(20.4)	46	(13.5)	0.02
Maternal death	0	(0.0)	4	(0.4)	0.36	3	(1.1)	3	(0.9)	0.78
Adverse maternal outcome^3^	88	(41.1)	793	(79.1)	<0.001	160	(59.3)	259	(76.2)	<0.001

^1^ Liters; ^2^ given with median and interquartile range; ^3^ composite of hysterectomy, mortality or total volume of blood loss ≥ 2,5L

### 3.3 Initial PPH-management

We do not report any statistically significant difference in terms of initial PPH-management among women with severe PPH between both countries. After vaginal birth 677/1002,67.5 % of women with severe PPH in the Netherlands received a first-line uterotonics versus 151/214,71.9 %, p = 0.39 in France and 231/342,67.5 % versus 192/270,71.1 % respectively (p = 0.34) after cesarean. See (Table 3).

**Table 3:** Univariate analysis. Comparing first-line management of PPH in women with severe PPH stratified by mode of birth between France and the Netherlands (2011–2013).

	Vaginal Birth					Cesarean birth				
	France N = 214		The Netherlands N = 1002		P-value	France N = 270		The Netherlands N = 342		P-value
Manual removal placenta	65	(30.4)	328	(32.8)	0.50	232	(85.9)	301	(88.0)	0.44
Uterine exploration after spontaneous birth placenta	149	(69.6)	658	(65.8)	0.26	30	(11.1)	36	(10.5)	0.81
Urinary catheterization	193	(90.1)	932	(93.0)	0.15	270	(1 0 0)	342	(1 0 0)	1
Oxytocin infusion^1^	151	(71.9)	677	(67.5)	0.39	192	(71.1)	231	(67.5)	0.34
Time PPH diagnosis – oxytocin infusion (median (IQR)) (minutes)	5	(0–15)	7 (0–18)		0.42	0 (0–5)		1 (0–7)		0.64

^1^In France, Oxytocin 5–10 IU slow IV followed by oxytocin infusion of 5–10 IU/h for 2 h (Max: 40 IU). In the Netherlands: Oxytocin 5 IU slow IV, followed by oxytocin infusion 2.5 IE/4h. PPH = postpartum hemorrhage. IQR = interquartile range

### 3.4 Use and timing of resuscitation and transfusion management

Table 2 describes resuscitation/transfusion management per country. Fibrinogen was given less frequently in the Netherlands than France regardless mode of birth (vaginal birth: 83/1002,8.3 % versus 105/2014,49.5 %, p < 0.001 respectively; cesarean: 47/342,13.7 % and 152/270,55.6 %, p < 0.001). Tranexamic acid and rVIIa were significantly less used after vaginal birth in the Netherlands than France (42.7 %, 428/1002 versus 55.6 %, 119/214, p < 0.001) and (2.3 %, 23/1002 versus 6.5 %, p < 0.001) respectively. Time to transfusion after PPH-onset was similar among countries (Table 4).

**Table 4:** Univariate analysis. Comparing transfusion therapy in women with severe PPH stratified by mode of birth between France and the Netherlands (2011–2013).

	Vaginal birth				Cesarean birth					
	France N = 214		The Netherlands N = 1002			France N = 270		The Netherlands N = 342		
	n	(%)	n	(%)	P-value	n	(%)	n	%)	P-value
Volume replacement therapy *Crystalloids* *Colloids* *Crystalloids and colloids*	198 19 19 158	(92.5) (9.1) (9.4) (74.0)	960 95 49 816	(95.8) (9.5) (4.9) (81.4)	0.04	250 30 4 216	(92.5) (11.1) (1.4) (80.0)	303 27 0 276	(88,6) (7.8) 0 (80,8)	0.07
Blood transfusion										
Red blood cells Number of units^1^	214 4	(100,0) (3–7)	1002 4	(100.0) (3–5)	1	270 5	(100.0) (3–7)	342 4	(100.0) (3–6)	1
Fresh frozen plasma Number of units^1^	196 2	(92.5) (2–4)	876 2	(87.4) (2–3)	0.04	243 4	(90.0) (2–6)	301 2	(88.9) (2–5)	0.91
Thrombocytes Number of units^1^	44 1	(20.9) (1–2)	185 1	(18.5) (1–2)	0.4	64 1	(23.7) (1–3)	110 1	(32.8) (1–2)	0.01
Hemostatic agents										
Fibrinogen	105	(49.5)	83	(8.3)	< 0.001	150	(55.6)	47	(13.7)	< 0.001
Tranexamic acid	119	(55.6)	428	(42.7)	< 0.001	140	(52.3)	167	(50.1)	0.51
Factor VII	14	(6.5)	23	(2.3)	< 0.001	6	(2.2)	14	(6.5)	0.23
Time between PPH^2^ diagnosis and start blood transfusion (median (IQR^3^)) (minutes)	95	(48–159)	95	(60–162)	0.94	73	(26–199)	80	(35–130)	0.35

^1^ among those who were transfused, ^2^ postpartum hemorrhage, ^3^ inter quartile range.

### 3.5 Use and timing of second-line uterotonics

Practitioners in both countries primarily applied sulprostone as second-line uterotonic. These were started significantly later after PPH-onset in the Netherlands than France (vaginal birth: 46 versus 25 min,p < 0.001; cesarean: 45 versus 18 min,p < 0.001) (Table 5).

**Table 5:** Univariate analysis. Comparing second-line therapy and uterine-sparing interventions in women with severe PPH stratified by mode of birth between France and the Netherlands (2011–2013).

			Vaginal birth			Cesarean birth				
	**France**		**The Netherlands**			**France**		**The Netherlands**		
	N = 214		N = 1002			N = 270		N = 342		
Second-line uterotonics	N	(%)	N	(%)	P-value	N	(%)	N	(%)	P-value
Administered *Ergot alkaloids* *Sulprostone* *Misoprostol*	187 0 178 9	(87.3) (0.0) (83.1) (4.2)	826 99 679 408	(82,4) (9,9) (67,7) (40,7)	0.03	192 0 190 2	(71.1) (0,0) (70.3) (0.7)	236 28 221 83	(69.0) (8.2) (64.6) (24.2)	0.35
Time PPH^1^ diagnosis - second-line uterotonics (median (IQR^2^)) (minutes)	25	(15–45)	46	(17–90)	< 0.001	18	(8–45)	45	(12–113)	<0.001
Uterine sparing interventions										
Women with any uterine-sparing intervention	134	(62.6)	394	(39.3)	< 0.001	155	(57.4)	133	(38.9)	<0.001
Multiple uterine-sparing interventions	28	(13.1)	85	(8.4)	0.03	23	(8.5)	37	(10.8)	0.30
Intra uterine tamponade	86	(40.2)	347	(34.6)	0.12	28	(10.4)	86	(25.2)	<0.001
Uterine compression sutures / vascular ligation	30	(14.1)	16	(1.6)	< 0.001	107	(39.6)	27	(7.9)	<0.001
Embolization	46	(21.5)	116	(11.6)	< 0.001	43	(15.9)	57	(16.7)	0.89

1 postpartum hemorrhage; ^2^ inter quartile range.

### 3.6 Use and timing of uterine-sparing interventions

After both vaginal and cesarean birth, patients with severe PPH were significantly (p < 0.001) less likely to be treated with uterine-sparing interventions in the Netherlands (vaginal birth: 394/1002, 39.3 % and cesarean 133/342, 38.9 %) versus France (vaginal birth: 134/214,62.6 % and cesarean 155/270,57.4 %)**.** After vaginal birth, 16/1002, 1.6 % of women with severe PPH were treated with vascular ligation or compression sutures in the Netherlands versus 30/214 (14.1 %) in France (p < 0.001) and 27/342 (7.9 %) against 107/270 (39.6 %) after cesarean (p < 0.001). Intra-uterine balloon tamponade was more commonly used in the Netherlands than France after cesarean (86/342,25.2 % versus 28/270,10.4 %, p < 0.001). Embolization was less frequently applied in the Netherlands than France after vaginal birth (116/1002, 11.6 % versus 46/214,21.5 %, p < 0.001) but similar in case of cesarean (Table 5).

**Fig. 1:**
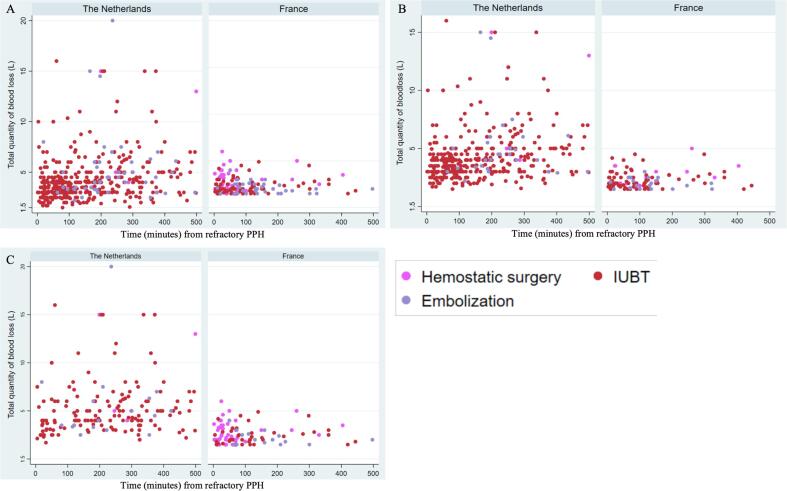
a-1c. Forest plot of total blood loss according to time of the first uterine-sparing intervention among women with refractory PPH in France and the Netherlands between 2011 and 2013 (a) regardless mode of birth (b) for vaginal birth (c) for cesarean birth.

Timing of application of the first uterine-sparing intervention was assessed among 1121 women with refractory PPH (severe PPH refractory to first-line management) in the Netherlands versus 422 women in France (Fig. S1) The total volume of blood loss in relation to the time of onset of the first uterine -paring intervention is displayed for all births and stratified by mode of birth in Fig. 1a-1c. In the Netherlands, all types of uterine-sparing intervention were initiated significantly later in the course of refractory PPH (Table 6) In the Netherlands, 101/1121,9% women received their first uterine-sparing intervention within the first hour after onset of refractory PPH versus 253/422,60 % in France (p < 0.001).

**Table 6:** Comparing median timing between onset of refractory PPH and application of uterine sparing-interventions among women with refractory PPH stratified by mode of birth between France and the Netherlands (2011–2013).

	Vaginal birth					Cesarean birth				
	France N = 188		The Netherlands N = 868		P-value	France N = 234		The Netherlands N = 253		P-value
	Median	IQR^1^	Median	IQR		Medan	IQR	Median	IQR	
Time^2^ between diagnosis refractory PPH^3^ - intra-uterine tamponade	39	28–115	59	55–220	0.02	45	30–150	95	(54–315)	< 0.001
Time between diagnosis refractory PPH - compression sutures / ligation	107	80–312	291	250–453	< 0.001	36	28–100	250	220–480	< 0.001
Time between diagnosis refractory PPH - embolization	127	113–322	206	180–387	< 0.001	152	115–380	270	199–485	< 0.001

1 = inter quartile range, ^2^ = time in minutes, ^3 =^ postpartum hemorrhage.

In total, 41/1121,3.6 % of women in the Netherlands had ≥ 8 L of blood loss versus zero women in France. The profile of these women is presented in Table S6.

## 4 Discussion

We report a higher incidence of adverse maternal outcome among women with severe PPH in the Netherlands as compared to France. Although there was no difference between countries in terms of the number of women with severe PPH receiving second-line uterotonics, we report a statistically significant longer delay before administration of second-line uterotonics in women with severe PPH in the Netherlands as compared to France. A larger proportion of women in France received hemostatic agents such as Fibrinogen. After both vaginal and cesarean birth, patients with severe PPH in France were significantly more likely to be treated with uterine-sparing interventions which were applied in an earlier stage of hemorrhage in France than the Netherlands.

The increased risk of adverse maternal outcome among women suffering severe PPH in the Netherlands as compared to France reported in this study, seems mainly due to the fact that more women suffered major obstetric blood loss in the Netherlands which is confirmed by the increased volumes of total blood loss among women in the Netherlands. This could perhaps be explained by a more expectant management in this country once initial management of PPH has failed, such as increased delay before administration of second-line uterotonics and uterine-sparing interventions (NVOG, 2018, Sentilhes et al., 2016). The consequences of such delay have also been demonstrated in a large Canadian cohort of vaginal deliveries, reporting a higher odds for hypotension and transfusion for every 5-minute additional delay in the administration of a second-line uterotonic (Knoll et al., 2022). The more frequent use of intra-uterine balloon tamponade and the low use of compression sutures and vascular ligation after cesarean in the Netherlands may also have contributed to the differences in adverse maternal outcome since intra-uterine balloon tamponade may take a longer time to stop the bleeding (Liu et al., 2021, Revert et al., 2017, Kong and To, 2018). Reported variations cannot be explained by different recommendations since both countries recommend surgical uterine-sparing interventions as a first step after failure of second-line uterotonics in case of cesarean. This stresses the need to investigate whether Dutch recommendations are interpreted differently or whether obstetricians in the Netherlands feel less inclined to perform hemostatic surgery and if so, why. In this context, surgical simulation trainings could be of interest (Kerbage et al., 2016). The implementation of specific PPH-care bundles as recommended by the World Health Organization could improve guideline adherence (WHO, 2023). Another explanation of the reported variations in terms of blood loss could be different methods of blood loss quantification applied in both studies. However, studies comparing the methods applied in both cohorts do not report any evidence to consider one method as more accurate over the other method (Diaz et al., 2018).

We report a higher use of hemostatic agents among women with severe PPH in France. It has been hypothesized that hypofibrinogemia is a marker of the risk of severe PPH suggesting early supplementation could reduce severity of PPH (Cortet et al., 2012). However, this hypothesis was not confirmed by recent studies, showing no reduction of blood loss or improved maternal outcomes after early and systematic treatment with fibrinogen. These findings make it unlikely that the differences in terms of fibrinogen use can explain the increased blood loss among women in the Netherlands and emphasize the need for more prospective and randomized trials to define optimal transfusion strategies among obstetric patients (Deleu et al., 2022).

In contrast with previous data from national obstetric surveillance systems, which reported a hemorrhage related maternal mortality ratio double as high for France as the Netherlands (0.9 (95 % CI 0.5–1.3) versus 0.4 per 100,000 livebirths (95 % CI 0.0–1.0)), we did not find any significant difference in terms of hemorrhage related maternal mortality in this study (Diguisto and Saucedo, 2022, Ducloy-Bouthors et al., 2021). This finding could perhaps be explained in two directions: the fact that the Epimoms study was not nationwide, or by an underreporting of maternal deaths in the Dutch national obstetric surveillance system due to the absence of crosslinking, which has previously resulted in underestimation of maternal deaths in the Netherlands (Ramler et al., 2022, Kallianidis et al., 2018).

The aforementioned findings could be interpreted as a reason to escalate management sooner rather than late. Also, among the maternal deaths reported in this study we reported marginal use of uterine sparing interventions. In line with other data, in the Netherlands 4 out of 7 women died with the uterus still preserved, stressing the importance of timely escalation of management. Nevertheless, our study findings also stress to remain vigilant to the overuse of uterine sparing interventions. Indeed, 40 % of the French women not meeting our inclusion criteria were treated by a uterine-sparing intervention which was initiated almost simultaneously with second-line uterotonics. In line with the high hysterectomy rate reported in this study in France, this may suggest that obstetricians in France escalate very rapidly when it comes to PPH-management, exposing women to the downsides of such escalation. Earlier studies reported a 9-fold increased risk for hysterectomy among women who gave birth by cesarean yet by stratifying our analyses to mode of birth we were able to bring to light that the reported differences in terms of hysterectomy between both countries cannot be explained by the fact that more women in France gave birth by cesarean (Kallianidis et al., 2020).

The reported incidences of severe PPH in France and the Netherlands in this study are difficult to compare with other high-income countries given the heterogeneity of the applied definitions of severe PPH among studies, which are compounded by the lack of a uniform and global definition of severe PPH (Prick et al., 2015, Kramer et al., 2013, Pettersen et al., 2023, Borovac-Pinheiro et al., 2018). Perhaps these differences could be explained by the reported variations in terms of mode of birth or by differences in PPH risk-factors such as hypertensive disorders and history of PPH. The higher proportion of women with vaginal birth in the Netherlands may have resulted in increased barriers and delays before escalation to more invasive management. This may also be compounded by several elements pertaining to maternity care in this country. First, the culture in the Netherlands might revolve more around the notion that physiological birth should proceed without unnecessary interventions, a notion strongly present among parturients and practitioners (Johnson et al., 2007). Second, differences in clinical practices with regards to manual removal of the placenta between France and the Netherlands may contribute to the delay in the Netherlands before proceeding to more invasive interventions. In the Netherlands, women are generally transferred to the operating theatre for general anesthesia for manual removal of the placenta whereas in France this is performed at the labor ward with the epidural that was already in place for labor (Seijmonsbergen-Schermers et al., 2018, Cheung et al., 2011). The reported differences in terms of mode of delivery among women with severe PPH in both countries cannot be explained by differences in general cesarean section rates alone which were 16.6 % in the Netherlands versus 20.2 % in France during the study-period (Kerbage et al., 2016, WHO, 2023). They may reflect variations between specific targets in PPH management resulting in increased risk of severe PPH after cesarean in France such as suboptimal postoperative surveillance, as has been suggested by earlier reports from the French national confidential enquiry, or by an increased risk of PPH among women given birth vaginally in the Netherlands due to the specific elements pertaining to maternity care in the Netherlands specified above.

The increased number of women with hypertensive disorders and history of PPH in the Netherlands may warrant a more proactive approach of these women, as has been highlighted by earlier studies (Schaap et al., 2019, Zwart et al., 2008). Another explanation could be differences in initial management of hemorrhage, leading to more women with severe PPH in the Netherlands. This seems however to be an unlikable explanatory mechanism as we did not report any differences in terms of prophylactic and initial management of hemorrhage.

This study is one of few comparing extensive data on timing of PPH management and maternal outcome in women with equally severe PPH from two countries with comparable resources. Both countries provided detailed information on the timing of interventions, an important determinant of PPH-related maternal outcome. Setting up a randomized controlled trial to test the efficacy of a therapeutic sequence in the treatment of refractory PPH is very challenging; in this context, international comparisons between countries with different management strategies offer a valuable quasi-experimental alternative for generating evidence. Although the EpiMOMs cohort was not nationwide, the large source population had characteristics similar to the national profile. Both databases were collected over comparable time-periods, reducing the probability that differences in outcome are the result of temporal trends in maternal characteristics. Main limitations arise from the fact that clinical practices may have changed since data from both the cohorts were collected. However, guidelines from both countries did not have any major revisions during the study-period besides from the use of intra-uterine balloon tamponade as a bridging step after vaginal birth in the 2014 revision of the French guideline. Although this could have resulted in an underuse of intra-uterine balloon tamponade in France in our study as compared to current practices, we consider the implementation of guidelines as a continuous process making it likely that this tool was already current practice among practitioners in 2012–2013. From the French source population, we excluded 79 women due to missing data on the total quantity of blood loss or blood transfusion. From these women, 24 had an invasive intervention to treat PPH and the fact that these women could not be included in our analyses could potentially have induced a selection bias.

## 5 Conclusions

We report a higher incidence of adverse maternal outcome among women with severe PPH in the Netherlands as compared to France. This difference could not be explained by differences in terms of patient characteristics or initial management of PPH but may be explained by the finding that uterine sparing interventions to treat severe PPH were applied more frequently and earlier after diagnosis of refractory hemorrhage in France than in the Netherlands. It may also be explained by the finding that patients in France were more likely to have an operative or cesarean delivery than those in the Netherlands, allowing for more expeditious access to uterine sparing interventions and hysterectomy.

## CRediT authorship contribution statement

**P.L.M. de Vries:** Conceptualization, Data curation, Formal analysis, Investigation, Methodology, Validation, Visualization, Writing – original draft. **C. Deneux-Tharaux:** Writing – review & editing, Validation, Supervision, Methodology, Conceptualization. **C. Caram:** Writing – review & editing, Visualization, Validation, Methodology, Formal analysis, Data curation. **F. Goffinet:** Writing – review & editing, Validation, Methodology, Conceptualization. **D.D.C.A. Henriquez:** Data curation, Validation, Writing – review & editing. **A. Seco:** Visualization, Validation, Methodology, Data curation. **J.G. van der Bom:** Writing – review & editing, Validation, Methodology, Conceptualization. **T. van den Akker:** Conceptualization, Writing – review & editing, Supervision, Validation, Methodology.

## Declaration of competing interest

The authors declare that they have no known competing financial interests or personal relationships that could have appeared to influence the work reported in this paper.

## Data Availability

Data will be made available on request.
