# Supplementary material for: Severe postpartum hemorrhage and the risk of adverse maternal outcome: A comparative analysis of two population-based studies in France and the Netherlands
Source: Prev Med Rep. 2024 Feb 23;40:102665. doi: 10.1016/j.pmedr.2024.102665 (PMC10907197; doi:10.1016/j.pmedr.2024.102665)
Supplement: Supplementary data 1 [file mmc1.docx]

**Supporting information**

Table S1. Comparative overview of national guidelines on PPH in France and the Netherlands applicable during the study-period (2011-2013)

Table S2. Availability and comparability of variables from each respective dataset with women with severe PPH in the Netherlands and France

Table S3: Overview of the number of patients with missing values per variable among the included women with severe PPH in the French and the Dutch dataset.

Table S4. Primary underlying cause of bleeding in women with severe PPH stratified by mode of birth in France and the Netherlands (2011-2013)

Table S5: Characteristics of the women who died from severe PPH in France and the Netherlands (2011-2013).

Table S6: Characteristics of women with severe PPH having extreme blood loss (>8L) in the Netherlands (2011-2013) (2011

**Figure S1**

**Original definition severe PPH TEMPOH-1 and EPIMOMS study:**

TEMPOH-1: Women who received either ≥4 units of RBC or a multicomponent blood transfusion within 24 h following birth because of postpartum hemorrhage exceeding 1000 mL

EPIMOMS: blood loss ≥ 1500 mL; and/or hemorrhage with (blood transfusion ≥ 4 RBC or embolization or vascular ligation or compressive uterine suture or Emergency peripartum hysterectomy or any organ dysfunction)

**Definitions used in this study:**

Harmonized definition severe PPH: blood loss ≥ 1500 ml AND (transfusion ≥4 units of RBC OR a multicomponent blood transfusion) within 24 h following birth

Harmonized definition refractory PPH**:** Women with severe PPH according to our harmonized definition for severe PPH, refractory to first-line management. First-line management consisted ofuterine massage, exploration of the uterine cavity, assessment of the genital tract and administration of oxytocin.
