# Supplementary material for: Severe postpartum hemorrhage and the risk of adverse maternal outcome: A comparative analysis of two population-based studies in France and the Netherlands
Source: Prev Med Rep. 2024 Feb 23;40:102665. doi: 10.1016/j.pmedr.2024.102665 (PMC10907197; doi:10.1016/j.pmedr.2024.102665)
Supplement: Supplementary data 2 [file mmc2.docx]

| Table S1. Comparative overview of national guidelines on PPH in France and the Netherlands applicable during the study-period (2011-2013) | | |
| --- | --- | --- |
|  | **France** | **The Netherlands** |
| Prophylactic management of PPH | | |
| Prophylactic uterotonics | Oytocin: 5 or 10 IU IM or slow IV.  Cesarean: 5–10 IU slow IV followed by IV infusion not exceeding 10 IU/h. | Oxytocin: 5 IU IM or slow IV.  Cesarean/women at increased risk of PPH: 5 IU slow IV followed by IV infusion 10 IU/4h. |
| Controlled cord traction | Not recommended | Recommended |
| Early cord clamping | Not recommended | Not recommended |
| Manual removal of placenta in the absence of bleeding | Between 30 and 60 min after birth | Between 30-60 min after birth; 60 minutes if blood loss < 500ml, vital parameters normal and operating theatre immediately available. |
| Initial management of PPH | | |
| First-line uterotonic | Oxytocin 5-10 IU slow IV or IM followed by oxytocin infusion 5–10 IU/h for 2 h (Max: 40 IU) | Oxytocin 5 IU slow IV, followed by oxytocin infusion 2.5 IU/4h |
| Measurement of blood loss | Collector bag | Weigh blood loss |
| Uterine massage | Recommended | Recommended |
| Emptying bladder | Recommended | Not specified |
| Manual uterine exploration | Recommended | Not specified |
| Second-line uterotonics | | |
| Misoprostol | Not recommended | Not recommended |
| Injectable prostaglandins | Sulprostone maximum 500 mcg in 500ml/h | Sulprostone 500 mcg / 30 min, maintenance dose 60-120 mcg/h |
| Oxytocin agonist | Not recommended | Not recommended |
| Ergot alkaloids | Not recommended | Ergometrine 0.2 mg IV or IM |
| Uterine sparing interventions | | |
| Uterine tamponade | Use left to the clinician’s choice after failure of second-line uterotonic and before recourse to surgical or interventional radiology management.  Method: intra-uterine balloon tamponade. | Recommended after failure of second-line uterotonics.  Method: intra uterine balloon tamponade. |
| Radiological arterial embolization | Recommended in case of vaginal birth in hemodynamically stable patients after failure of second-line uterotonics. | Recommended in case of vaginal birth after failure of uterine tamponade. |
| Compression sutures | Recommended in case of vaginal birth in hemodynamically unstable patients after failure of second-line uterotonics. Recommended ain case of caesarean after failure of second-line uterotonics.  Type: No type is recommended over another. | Recommended in case of cesarean after failure of second-line uterotonics.  Type: B-Lynch. |
| Vascular ligation | Recommended in case of vaginal birth in hemodynamically unstable patients after failure of second-line uterotonics. Recommended in case of caesarean after failure of second-line uterotonics  Type: Uterine artery ligation/internal iliac artery ligation | Optional but embolization considered as effective.  Type: Uterine artery ligation / internal iliac artery ligation |
| Last resort management | | |
| Hysterectomy | After failure of uterine sparing interventions. Surgical approach in function of surgeon’s preference | In case of life-threatening bleeding. Surgical approach not specified. |
| Transfusion management | | |
| Fluid replacement therapy | Crystalloids | Crystalloids |
| Erythrocyte concentrates | Goal directed transfusion.  Maintain hemoglobine concentration $\geq$8 g/dL. | Fixed ratio transfusion.  No specific guideline for PPH. Guideline refers to general national transfusion protocol which recommends a ratio from FFP: RBC of 1:2 |
| FFP | Depending on the severity of the haemorrhage or coagulopathy | See above, usually maintaining a ratio from FFP:RBC of 1:2 |
| Thrombocytes | Maintain above 50 × 10^9^/l | Not specified |
| Cell Salvage | Not specified | Not specified |
| rFVIIa | Not recommended, only for an uncontrolled haemorrhage after failure of conventional treatment and after having attempted to correct platelet levels and other haemostasis indicators | Not recommended but may be considered after consultation of specialist in massive haemorrhage |
| Fibrinogen | Maintain $\geq$ 2 g/L | Maintain $\geq$ 2.5 g/L. |
| Tranexamic acid | Left to clinicians’ discretion | Left to clinicians’ discretion |
| PPH = postpartum hemorrhage, IU: international units, IM = intramuscular, IV = intravenous, h= hour, ml= Milliliters, mcg= microgram, mg= milligram, dL= deciliter, l= liter, FFP = fresh frozen plasma, RBC= red blood cells, rFVIIa= recombinant factor VIIa | | |

**Table S2. Availability and comparability of variables from each respective dataset with women with severe PPH in the Netherlands and France**

| **Desired variable** | **France (EPIMOMS)** | **the Netherlands (TeMpOH-1)** | **Included in analysis** | **Comment** |
| --- | --- | --- | --- | --- |
| Age | *Yes* | *Yes* | Yes |  |
| Obestiy | *Yes* | *Yes* | Yes |  |
| Tabagism | *Yes* | *No* | No |  |
| History of PPH | *Yes* | *Yes* | Yes |  |
| Polyhydramnios | *No* | *No* | No |  |
| Hypertensive disorder | *No* | *Yes* | Yes | *New variable created in Epimoms: hypertensive disorder created as a composite of preeclampsia + help + chronic hypertension +* |
| Multiple pregnancy | *Yes* | *Yes* | Yes |  |
| Macrosomia | *No* | *No* | No | *Birth weight available. Variable macrosomia defined in both databases as birth weight > 4000 gr at birth* |
| *Characteristics of birth* |  |  |  |  |
| Parity | *Yes* | *Yes* | Yes |  |
| Previous caesarean section | *Yes* | *Yes* | Yes |  |
| Mode of birth | *Yes* | *Yes* | Yes |  |
| Weeks gestational age | *Yes* | *Yes* | *Yes* |  |
| Induction of labor | *Yes* | *Yes* | *Yes* |  |
| Analgesics during labor | *No* | *Yes* | *No* |  |
| Mode of birth | *Yes* | *Yes* | *Yes* |  |
| Infection during labor | *No* | *Yes* | *No* |  |
| Primary cause of the bleed | *Yes, see comment* | *Yes* | *Yes* | *We used a hierarchical approach as to maintain one primary cause: abnormal placentation, placenta previa, placental abruption, trauma, other, atony, retained placenta.* |
| *Third stage of labor* |  |  |  |  |
| Prophylactic oxytocin | *Yes* | *Yes* | *yes* |  |
| Exploration of the genital tract | *No* | *Yes* | *No* |  |
| Manual removal of the placenta | *Yes* | *Yes* | *yes* |  |
| Manual revision of the uterine cavity | *Yes* | *Yes* |  |  |
| Time diagnosis PPH | *Yes* | *Yes* | *yes* |  |
| *Transfusion therapy* |  |  |  |  |
| Colloid (ml) | *Yes* | *Yes* | *Yes* |  |
| Crystalloid (ml) | *Yes* | *Yes* | *Yes* |  |
| Number of units of RBC | *Yes* | *Yes* | *Yes* |  |
| Fresh Frozen Plasma | *Yes* | *Yes* | *Yes* |  |
| Number of units of fresh frozen plasma | *Yes* | *Yes* | *Yes* |  |
| Platelets | *Yes* | *Yes* | *Yes* |  |
| Number of units of platelets | *Yes* | *Yes* | *Yes* |  |
| Fibrinogen | *Yes* | *Yes* | *Yes* |  |
| Cell Saver | *No* | *No* | *No* |  |
| Factor VIIa | *Yes* | *Yes* | *Yes* |  |
| Tranexamic acid | *Yes* | *Yes* | *Yes* |  |
| Timing of first transfusion unit | *Yes* | *Yes* | *Yes* |  |
| Delay first transfusion unit and PPH | *No, see comment* | *No, see comment* | *Yes* | *Variable created by substracting timing first transfusion unit – time diagnosis PPH* |
| *Second-line uterotonics* |  |  |  |  |
| Ergot alkaloids | *Yes* | *Yes* | *Yes* |  |
| Sulprostone | *Yes* | *Yes* | *Yes* |  |
| Misoprostol | *Yes* | *Yes* | *Yes* |  |
| Timing first second-line uterotonics | *Yes* | *Yes* | *Yes* |  |
| Delay second-line uterotonics and diagnosis PPH | *No, see comment* | *No, see comment* | *Yes* | *Variable created by substracting timing start second-line uterotonics– time diagnosis PPH* |
| *Invasive interventions* |  |  |  |  |
| Any invasive intervention | *No* | *No* | *No* | *Variable created as a composite of (intra-uterine balloon tamponade yes/no, conservative surgery yes/no)* |
| Intra uterine tamponade | *Yes* | *Yes* | *Yes* |  |
| Conservative surgery | *No, see comment* | *Yes* | *Yes* | *Created as a variable of B-lynch + vascular ligation* |
| Embolization | *Yes* | *Yes* | *Yes* |  |
| Multiple invasive interventions | *Yes* | *Yes* | *Yes* |  |
| Timing each intervention | *Yes* | *Yes* | *Yes* |  |
| *Maternal morbidity* |  |  |  |  |
| Total volume of blood loss | *Yes* | *Yes* | *Yes* |  |
| Acute kidney failure | *No* | *Yes* | *No* |  |
| Intensive care unit admission | *Yes* | *Yes* | *No* | *Not included as criteria for ICU admission varied between countries making it an inappropriate variable to compare between countries* |
| Blood loss $\geq$ 2.5L | *No* | *No* | *No* | *Variable created by using total quantity of blood loss* |
| Transfusion $\geq$ 5PC | *No* | *No* | *No* | *Variable created by using total quantity of PC* |
| Hysterectomy | *Yes* | *Yes* | *No* |  |
| Maternal death | *Yes* | *Yes* | *Yes* |  |
| Adverse maternal outcome | *No, see comment* | *No, see comment* | *Yes* | *Variable created as a composite of total volume of blood loss* $\geq$*2.5L, hysterectomy or mortality* |
| *PPH= postpartum hemorrhage, L= liters, PC = packet cells* | | | | |

| Table S3: Overview of the number of patients with missing values per variable among the included women with severe PPH in the French and the Dutch dataset | | |
| --- | --- | --- |
| Missing values | France | The Netherlands |
| Parity | 5 | 0 |
| Gestational age at birth | 2 | 6 |
| Hypertensive disorder | 1 | 0 |
| Volume replacement therapy | 20 | 10 |
| FFP | 2 | 0 |
| Thrombocytes | 2 | 3 |
| Tranexamic acid | 6 | 7 |
| Factor VII | 1 | 3 |
| Second-line uterotonics | 7 | 0 |
| Intra uterine balloon tamponade | 4 | 0 |
| Embolization | 12 | 0 |
| Hysterectomy | 12 | 0 |

|  |  |  |  |  |  |  |  |  |  |  |  |  |
| --- | --- | --- | --- | --- | --- | --- | --- | --- | --- | --- | --- | --- |
| Table S4. Primary underlying cause of bleeding in women with severe PPH stratified by mode of birth in France and the Netherlands (2011-2013) | | | | | | | | | | |  |  |
|  | |  | **Vaginal birth** | | | **Cesarean birth** | | | | |  |  |
|  | | France N=214 |  | The Netherlands N=1002 |  | France  N=270 | |  | | The Netherlands  N=342 | |  |
|  | | n | (%) | n | (%) | n | (%) | n | | %) | |  |
| Atony | | 167 | (78,0) | 685 | (68) | 111 | (41,1) | 189 | | (55.3) | |  |
| Placenta Praevia | | 1 | (0,5) | 2 | (0.2) | 17 | (6,3) | | 17 | (5.0) | |  |
| Abnormally invasive placenta | | 7 | (3,3) | 68 | (6.8) | 30 | (11.1) | | 39 | (11.4) | |  |
| Placenta abruption | | 1 | (0,5) | 10 | (1.0) | 34 | (12,6) | | 14 | (4.1) | |  |
| Cesarean surgical injury | | 11 | (5,1) | 11 | (1.1) | 59 | (21.9) | | 76 | (22.2) | |  |
| Retained placenta | | 18 | (8,4) | 223 | (22.3) | 1 | (0.4) | | 4 | (1.2) | |  |
| Other^1^ | | 8 | (3,7) | 3 | (0.3) | 17 | (6.3) | | 3 | (0.9) | |  |
| Missing | | 1 | (0,5) | 0 |  | 1 | (0.4) | | 0 | | |  |
| ^1^ sepsis, diffuse intravascular coagulation, Willebrand disease, vasa praevia, myoma previa  France had multiple causes of PPH coded. We used a hierarchical approach as to maintain one primary cause. The hierarchy we defined was: abnormal placentation, placenta previa, placental abruption, trauma, other, atony, retained placenta. | | | | | | | | | | | | |
|  |  |  |  |  |  |  |  |  |  |  |  |  |

| Table S5: Characteristics of the women who died from severe PPH in France and the Netherlands (2011-2013) | | | | |  |
| --- | --- | --- | --- | --- | --- |
|  |  | | France  N=3 | | Netherlands  N=7 |
| Obesity (BMI > 30 kg/m2) |  | | 1 | | 1 |
| Parity |  | |  | |  |
| *Nulliparity* |  | | 1 | | 3 |
| *multiparous without previous cesarean* |  | | 1 | | 4 |
| *multiparous with previous cesarean* |  | | 1 | | 0 |
| History of PPH |  | | 0 | | 0 |
| Multiple pregnancy |  | | 0 | | 0 |
| Pre-eclampsia or HELLP |  | | 1 | | 2 |
| Macrosomia |  | | 0 | | 0 |
| Characteristics of birth |  | |  | |  |
| Induction of labour |  | | 1 | | 2 |
| Mode of birth  *Spontaneous vaginal birth*  *Instrumental delivery*  *Elective cesarean*  *Emergency cesarean* |  | | 0  1  1  1 | | 1  3  0  3 |
| Characteristics of hemorrhage |  | |  | |  |
| Cause of bleeding |  | |  | |  |
| *Atony* |  | | 2 | | 5 |
| *Placenta Praevia* |  | | 0 | | 0 |
| *Abnormally invasive placenta* |  | | 0 | | 0 |
| *Placenta abruption* |  | | 0 | | 0 |
| *Cesarean surgical injury* |  | | 1 | | 1 |
| *Retained placenta* |  | | 0 | | 1 |
| Maternal outcome |  | |  | |  |
| Median total volume of blood loss (L) |  | | 6 | | 12 |
| Hysterectomy |  | | 2 | | 3 |
| Second line uterotonics |  | |  | |  |
| *Sulprostone applied* | *3* | | |  | *7* |
| *Time PPH diagnosis - second-line uterotonics (median) (minutes)* | | *30 (20-40)* |  | | *90 (60-130)* |
| Uterine sparing interventions |  | |  | |  |
| *Intra-uterine tamponade* | *0* | |  | | *3* |
| *Embolization* | *0* | |  | | *1* |
| *Uterine compression sutures/ vascular ligation* | *2* | |  | | *1* |
| PPH= postpartum hemorrhage |  | |  | |  |

| Table S6: Characteristics of women with severe PPH having extreme blood loss (>8L) in the Netherlands (2011-2013) | | |
| --- | --- | --- |
|  |  | N=41 |
| Obesity (BMI > 30 kg/m2) |  | Too many missing data |
| Parity |  |  |
| *Nulliparity* |  | 12 (29.2) |
| *multiparous without previous cesarean* |  | 19 (65.5) |
| *multiparous with previous cesarean* |  | 10 (34.4) |
| History of PPH |  | 1 (3.4) |
| Multiple pregnancy |  | 5 (12.2) |
| Pre-eclampsia or HELLP |  | 4 (9.8) |
| Macrosomia |  | 3 (7.3) |
| Characteristics of birth |  |  |
| Induction of labour |  | 12 (29.3) |
| Mode of birth  *Spontaneous vaginal birth*  *Instrumental delivery*  *Elective cesarean*  *Emergency cesarean* |  | 11 (26.8)  9 (21.96)  10 (24.9)  11 (26.8) |
| ^1^ Women with extreme blood loss were defined as women with $\boldsymbol{\geq}$ 8 liters of blood loss (n=41), PPH= postpartum hemorrhage, BMI= body mass index | | |
