# Supplementary material for: Severe postpartum hemorrhage and the risk of adverse maternal outcome: A comparative analysis of two population-based studies in France and the Netherlands
Source: Prev Med Rep. 2024 Feb 23;40:102665. doi: 10.1016/j.pmedr.2024.102665 (PMC10907197; doi:10.1016/j.pmedr.2024.102665)
Supplement: Supplementary data 3 [file mmc3.pdf]

**Figure S1. Flowchart of patient inclusion from the French and the Dutch source population according to a harmonized definition of severe PPH.**

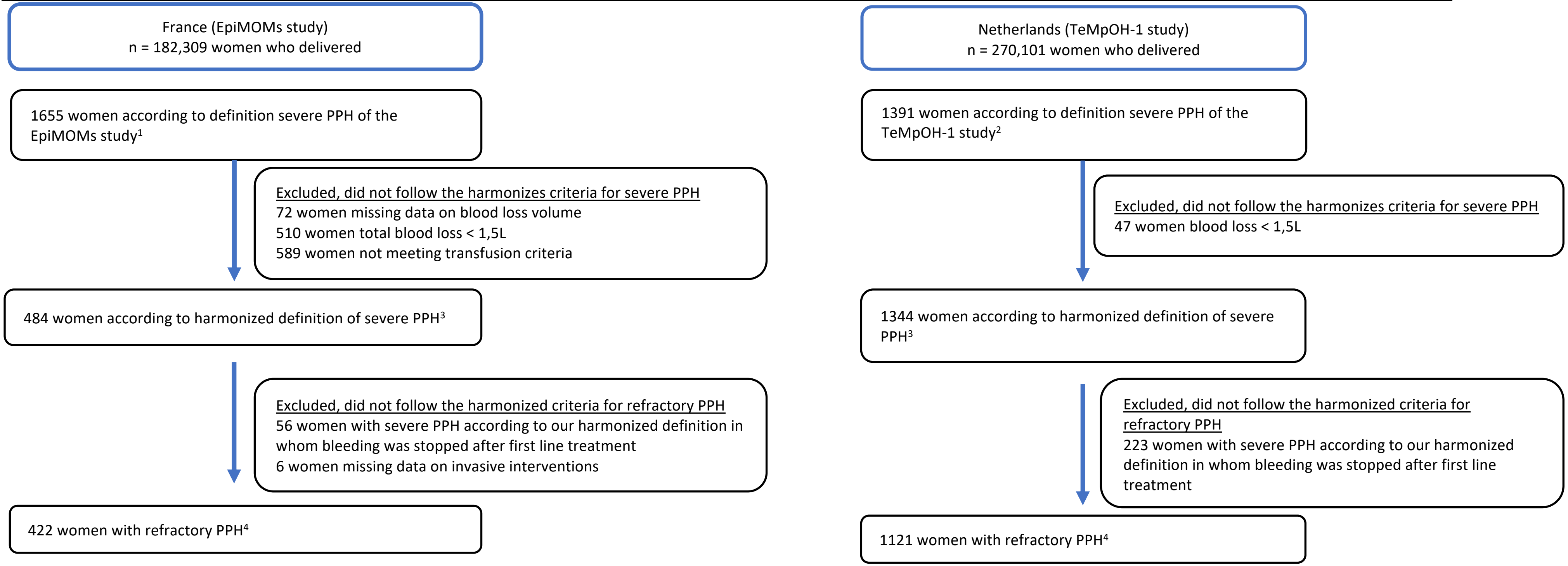


**Definitions used:**

[1] **TeMpOH-1 definition severe PPH:** women who received either ≥4 units of RBC or a multicomponent blood transfusion within 24 h following birth because of postpartum hemorrhage exceeding 1000 mL

[2] **EpiMOMs definition severe PPH**: blood loss ≥ 1500 ml; and/or hemorrhage with Blood transfusion ≥ 4 RBC or Arterial embolization or Vascular ligation or Compressive uterine suture or Emergency peripartum hysterectomy or any organ dysfunction (according to EpiMOMs definition)

[3] **Harmonized definition severe PPH**: blood loss ≥ 1500 ml AND transfusion ≥4 units of RBC OR a multicomponent blood transfusion within 24 h following birth

[4] **Refractory PPH**: severe PPH according to our harmonized definition, and which was refractory to first-line management (uterine massage, exploration of the uterine cavity, assessment of the genital tract and administration of oxytocin

PPH= postpartum hemorrhage, L=liters, RBC=red blood cells
